# CT findings in a patient with Coronavirus Disease-19-associated acute pericarditis

**DOI:** 10.1259/bjrcr.20210001

**Published:** 2021-04-09

**Authors:** Yuki Tashiro, Mana Kurihara, Yosiro Hori, Yuya Nakamura, Masaaki Kuarata, Tsutomu Toshida, Taku Asano, Mio Ebato, Hiroshi Suzuki, Toshi Hashimoto

**Affiliations:** 1 Department of Radiology, Showa University Fujigaoka Hospital, Yokohama, Japan; 2 Division of Cardiology, Department of Medicine, Showa University School of Medicine, Tokyo, Japan; 3 Division of Cardiology, Department of Internal Medicine, Showa University Fujigaoka Hospital, Yokohama, Japan

## Abstract

Coronavirus Disease-19 (COVID-19)-associated acute pericarditis is a rare complication. Several cases have been reported, but those reports have not discussed any imaging findings. Here, we report a case of a 76-year-old female diagnosed with COVID-19-associated pericarditis without pneumonia, and present image findings of the patient’s contrast-enhanced CT.

## Clinical presentation

A 76-year-old female presented with chest pain a week after mild upper respiratory tract symptoms occurred. She had a history of hypertension and Type II diabetes mellitus. Upon arrival at our outpatient clinic, physical examination revealed a blood pressure of 112/50 mmHg, heart rate of 100 beats per minute, oxygen saturation of 98% while breathing ambient air, body temperature of 37.2°C, and respiratory rate of 14 respirations per minute. The physical examination was abnormal concerning the pericardial friction rub at the apex. A 12-lead electrocardiogram showed low voltage in the precordial leads, s-wave in the Ⅰ lead, negative T-wave in the Ⅲ lead, and no evidence of ST elevation. Cardiomegaly and blunt costophrenic angles were evident in plain radiography of the chest. Laboratory examination revealed elevated levels of C-reactive protein [21.37 (normal range <0.3. mg/dL] and white blood cell counts [16,130 (normal range 3500–9700) mg dl^−1^] with 89.5% of neutrophils, 7.5% lymphocytes, and 3% mononuclear cells. Laboratory examination also revealed normal levels of markers of cardiomyocyte injury (high-sensitivity troponin I level <10.0 pg ml^−1^ and creatine kinase-MB level of 0.5 ng ml^−1^) and a slight increase in brain natriuretic peptide (BNP) levels of 77.0 pg ml^−1^.

## Differential diagnosis

Differential diagnosis included pericarditis related to conditions, such as infectious diseases (including tuberculosis), malignant tumors, and autoimmune diseases.

## Investigations

**Figure 1.**
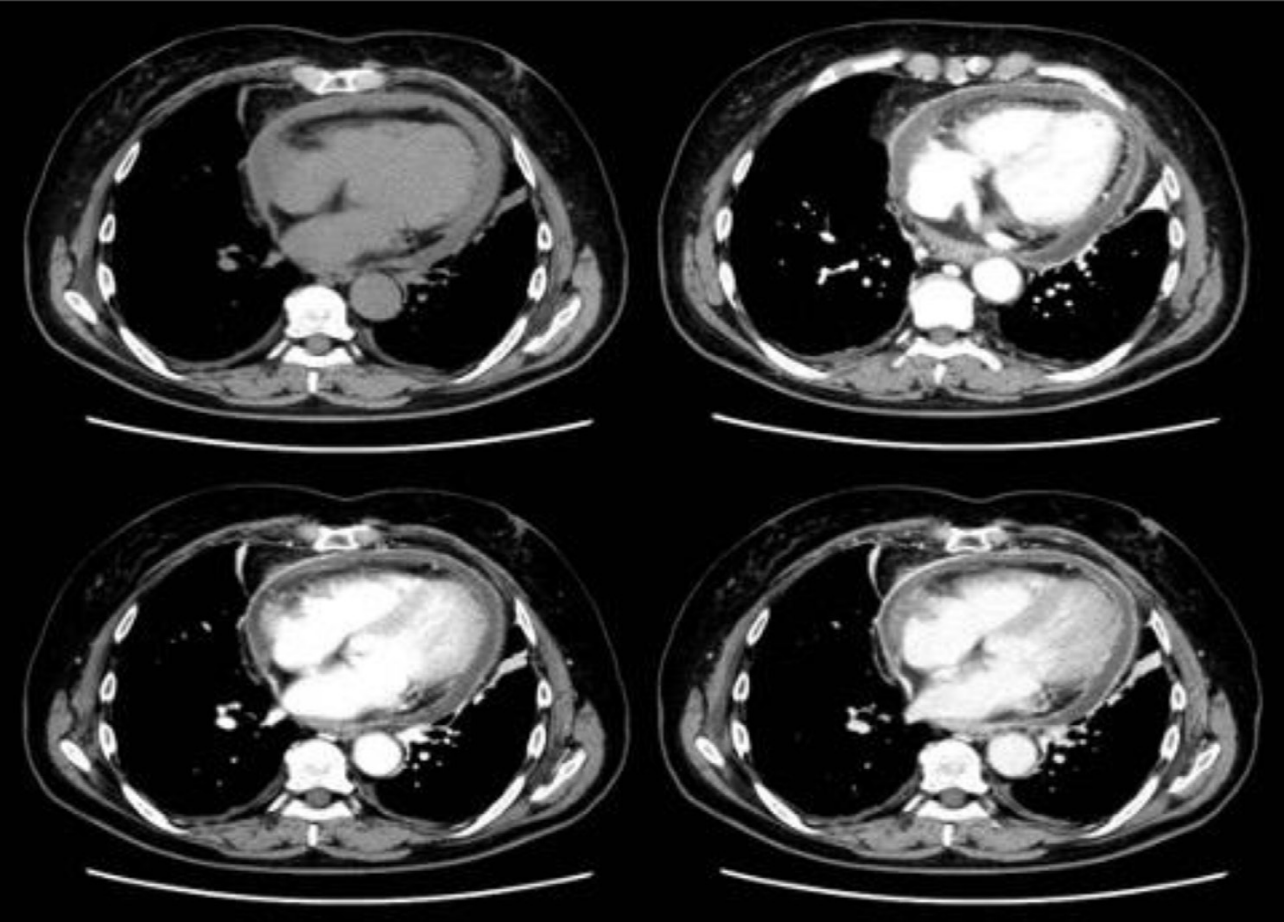
CT images revealed pericardium thickening and enhancement with a pericardial effusion that showed slightly high density compared with water (approximately 20 HU).

Contrast-enhanced CT images revealed pericardial thickening and enhancement with pericardial effusion that showed moderately high density (Figure 1). The chest CT images showed bilateral slight pleural effusion and subpleural atelectasis. There were no findings that indicated COVID-19 pneumonia. Transthoracic echocardiography revealed the presence of massive pericardial effusion, mild collapse of the right atrium during diastole and pericardial thickening. Since there were no findings suggestive of myocarditis, such as left ventricular systolic dysfunction or wall motion abnormalities, further examination with cardiac MRI was not performed.

## Management

The patient was admitted with a diagnosis of suspected pericarditis. Considering the possibility of prior coronavirus infection, she was quarantined in an isolation room. A nasopharyngeal swab was performed with a positive result for severe acute respiratory syndrome coronavirus 2 (SARS-CoV-2) in real-time reverse transcriptase polymerase chain reaction (RT-PCR) assay on Day 1. There was no etiological evidence of pericarditis, such as infectious diseases, including tuberculosis, malignant tumors, and autoimmune diseases. Transthoracic echocardiography, performed with the use of personal protective equipment on Day 1, revealed the presence of massive pericardial effusion and mild collapse of the right atrium during diastole and pericardial thickening. Under the impression of pericarditis without cardiac tamponade, oral administration of acetaminophen was initiated because of negative reports on the SARS-CoV-2 infection and oral non-steroidal anti-inflammatory drugs. Thereafter, the symptoms and slight fever soon subsided, and the patient was discharged 11 days later after two negative results for SARS-CoV-2 on real-time RT-PCR tests. At the time of discharge, plain radiography of the chest showed a normal heart size and a sharp costophrenic angle. Chest CT images also showed improvement of pericarditis findings.

## Discussion

The first cases of COVID-19 were reported in December 2019, originating in Wuhan, China, followed by an outbreak occurring all over the world. The disease symptoms resemble typical respiratory symptoms, and many findings of chest CT images have been reported.^1^ With an increase in reported cases, cardiac complications have become known, such as ischemic myocardial injury, myocarditis, and arrhythmia.^2^ Acute pericarditis is considered a relatively rare complication. There have been several case reports concerning COVID-19-associated pericarditis,^3–8^ but there is no report about CT imaging findings. In our patient, contrast-enhanced CT images revealed pericardial thickening and enhancement with a pericardial effusion that showed slightly high density compared with water (Figure 1). Pericardial thickening and enhancement in CT images suggest acute pericarditis with a sensitivity of 54–59% and a specificity of 91–96%,^9^ but the differential diagnosis widely range from inflammatory changes (infectious, autoimmune, and radiation) to neoplastic diseases.^10^ Several CT findings were reported to differentiate the various cause of pericarditis. For example, the irregularly thickened pericardium and the presence of malignant tumor may suggest the pericarditis related to malignancy.^11^ In practice, the CT findings of acute pericarditis due to various causes, including viral infections, are non-specific, and differentiation by imaging is considered to be often difficult. Raymond et al reported pericarditis with pediatric COVID-19 that was performed pericardiectomy.^12^ In this case, pericardium showed fibrinous thickening with neutrophil infiltration. In addition, some cases of COVID-19-associated pericarditis in which pericardiocentesis was performed reported its nature as serosanguinous to hemorrhagic. Although we did not perform pericardiectomy or pericardiocentesis, CT-findings may have reflected these inflammatory changes or fluid nature. There were no findings of pneumonia in the chest CT images. Karadeniz et al^3^ reported pericarditis with lung abnormalities, such as ground-glass opacification and subpleural curvilinear lines that were common findings in COVID-19 pneumonia in chest CT images. On the other hand, some reports have presented no abnormalities in chest plain radiographs.^5–8^ We considered that COVID-19-associated pericarditis should be included in the differential diagnosis in cases of acute pericarditis, even in the absence of obvious pneumoniae in chest CT images.

## Learning objective

To recognize that COVID-19-associated pericarditis should be included in the differential diagnosis in cases of acute pericarditis, even in the absence of obvious pneumonia.

CT findings of acute pericarditis associated with COVID-19 infection may present pericardial thickening and enhancement with pericardial effusion, and it was difficult to differentiate from pericarditis due to other causes.
